# Characterisation and stability of co-amorphous systems containing olanzapine and sulphonic acids

**DOI:** 10.1080/07853890.2021.1896098

**Published:** 2021-09-28

**Authors:** Inês A. Santos, Ana C. Bastos, João F. Pinto, Ana I. Fernandes

**Affiliations:** a Centro de Investigação Interdisciplinar Egas Moniz (CiiEM), Egas Moniz Cooperativa de Ensino Superior, Caparica, Portugal; b iMed.ULisboa – Research Institute for Medicines, Faculdade de Farmácia, Universidade de Lisboa, Lisboa, Portugal

## Abstract

**Introduction:**

A large number of active pharmaceutical compounds currently under development are poorly water soluble, which can limit their bioavailability and results in formulation challenges [1]. Co-amorphous systems (CAMs) are known to increase the apparent solubility and dissolution rate of drugs [1,2]. To date, sulphonic acids have never investigated as possible co-formers for Olanzapine (OLZ; a BSC class II drug) and, thus, the aim of this work is to evaluate their potential on the formation of stable CAMs.

**Materials and methods:**

OLZ was used as model drug. Saccharin (SAC), cyclamic acid (CA), acesulfame (ACE; obtained by neutralisation of potassium acesulfame) and their salts, sodium saccharin, sodium cyclamate and potassium acesulfame, respectively, were used as co-formers. Mixtures (2 g) of OLZ and each co-former, in molar ratios 1:1, were submitted to milling, solvent evaporation (SE) and quench cooling. Samples were characterised by differential scanning calorimetry (DSC), X-ray powder diffraction (XRPD), Fourier-transform infra-red spectroscopy (FTIR) and cooling/heating stage microscopy. Solubility assessment, powder dissolution rate and stability studies were also performed.

**Results:**

SAC, CA and ACE demonstrated to be successful co-formers in the production and stabilisation of OLZ-CAMs obtained by the three different techniques, presenting a single glass transition temperature (Tg) in thermograms and a typical halo in XRPD diffractograms. None of the salts were capable of forming a CAM, resulting in phase separation and absence of Tg events. Microscopical analysis supported DSC data and provided images of the CAMs recrystallization. Band shifts and broadening of the CAMs FTIR spectra suggest an intermolecular interaction between the N–H group in OLZ and the C = O group in SAC and ACE and the O-H group in CA. Solubility of OLZ was significantly increased (up to 199 times) when produced by SE with SAC. Dissolution rate was also increased for all the CAMs produced. SAC and CA successfully stabilised the CAMs produced, for more than 8 weeks at 25 °C/11, 53 and 75% RH and at 25 °C/11 and 53% RH, respectively.

**Discussion and conclusions:**

In this study OLZ was successfully amorphized using the neutral forms of the sulphonic acids. The impossibility of the salts to form a CAM is in agreement with the FTIR results, since the groups responsible for the molecular interaction with OLZ are unavailable in these molecules due to their negative charge. SE proved to be the best technique to produce CAMs with the different co-formers, resulting in the highest increase in solubility. SAC has shown to be the best co-former, with the highest solubility and stability over time, under higher relative humidity. Moreover, the increased dissolution rate of the CAMs suggests improved bioavailability of OLZ, a feature with therapeutic impact, which should be confirmed *in vivo*.

## References

[1] Blaabjerg LI, Lindenberg E, Rades T, et al. Influence of preparation pathway on the glass forming ability. Int J Pharm. 2017;521:232–239. 28232267 10.1016/j.ijpharm.2017.02.042

[2] Dengale SJ, Grohganz H, Rades T, et al. Recent advances in co-amorphous drug formulations. Adv Drug Del Rev. 2016;100:116–125. 10.1016/j.addr.2015.12.009 26805787

